# Resveratrol inhibits proliferation in human colorectal carcinoma cells by inducing G_1_/S-phase cell cycle arrest and apoptosis through caspase/cyclin-CDK pathways

**DOI:** 10.3892/mmr.2014.2406

**Published:** 2014-07-21

**Authors:** BIN LIU, ZHONGYOU ZHOU, WEI ZHOU, JIE LIU, QINGYU ZHANG, JUAN XIA, JUNTAO LIU, NIANPING CHEN, MINGYI LI, RUNZHI ZHU

**Affiliations:** 1 Laboratory of Hepatobiliary Surgery, Guangdong Medical College, Zhanjiang Key Laboratory of Hepatobiliary Diseases, Zhanjiang, Guangdong 524001, P.R. China; 2 Department of Radiology, Affiliated Hospital of Guangdong Medical College, Zhanjiang, Guangdong 524001, P.R. China

**Keywords:** apoptosis, cell cycle arrest, resveratrol, HCT116, caco-2

## Abstract

The present study compared the effect of resveratrol on HCT116 and Caco-2 human colon cancer cells. Annexin V/propidium iodide staining, MTT assay and western blot analysis revealed that resveratrol induced cycle arrest in the two cell lines, which was evidenced by cell cycle analysis and changes in the expression of the cell cycle proteins cyclin-dependent kinase (CDK) 2, CDK4, cyclin D1, proliferating cell nuclear antigen and P21. Furthermore, resveratrol was found to have a strong apoptosis-inducing effect, which was evidenced through the high percentage of annexin V positive cells and high protein expression of cleaved-caspase-7, cleaved-caspase-9 and cleaved-poly(ADP-ribose) polymerase in the resveratrol-treated cancer cells. In conclusion, these results demonstrated that resveratrol had greater growth inhibitory and cell cycle arrest effects on Caco-2 cells than HCT116 cells, through caspase-dependent and cyclin-CDK pathways.

## Introduction

**Figure 1:**
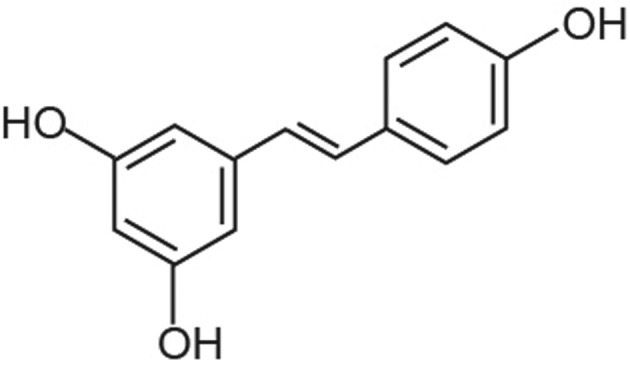
Chemical structure of resveratrol.

Resveratrol (Fig. 1) is a chemopreventive molecule which inhibits the proliferation of tumor cells of various etiologies. The biological properties of resveratrol have been described previously (1–3) and its beneficial effects have promoted the investigation of novel, more effective analogues (4,5). Resveratrol has been proposed to be a good anti-carcinogenic agent due to its low toxicity and capacity to modulate numerous molecular pathways involved in cancer progression (6). However, the direct molecular target of resveratrol remains elusive (7). Colon cancer is one of the types of cancer with the highest mortality rate in the United States and has been ranked the third most common cause of cancer mortality (8). Moreover, an increase in the colon cancer incidence in adults under 50 years of age has been reported (9). Cancer chemoprevention has been reported to be a promising strategy to prevent cancer death, particularly in colon cancer, due to the relatively slow progression of the colorectal adenomatous polyps in colon cancer, which enhances the opportunity for chemoprevention treatment. Results from epidemiological studies have associated fruit and vegetable consumption with a reduced risk of colon cancer, and various phytochemicals from commonly consumed fruit and vegetables have been identified as potential anticancer agents (10). The present study aimed to investigate the anti-cancer effect of resveratrol in HCT116 and Caco-2 human colon cancer cells. To the best of our knowledge, the present study is the first to demonstrate that caspase and cyclin-cyclin-dependent kinase (CDK) proteins are involved in resveratrol-induced apoptosis and cell cycle arrest.

## Materials and methods

### Drugs and reagents

Resveratrol was purchased from Sigma-Aldrich (St. Louis, MO, USA). Resveratrol was dissolved to a concentration of 50 mM in 100% dimethyl sulfoxide (DMSO) as a stock solution and stored at −20°C. The final DMSO concentrations used in the present study were ≤0.1%. Antibodies against cleaved-caspase-9, cleaved-caspase-7, cleaved-PARP, CDK2, CDK4, Cyclin D1, PCNA and GAPDH were all purchased from Cell Signaling Technology, Inc. (Beverly, MA, USA) and goat anti-rabbit immunoglobulin G (IgG)-horseradish peroxidase (HRP; EarthOx, LLC, San Francisco, CA, USA) was used as a secondary antibody.

### Cell culture and resveratrol treatment

HCT116 and Caco-2 human colon cancer cells were provided by the Affiliated Hospital of Guangdong Medical College (Zhanjiang, China). The HCT116 and Caco-2 cells were cultured in RPMI-1640 (Gibco-BRL, Grand Island, NY, USA) and McCoy’s 5A medium (Gibco-BRL), respectively, supplemented with 10% (v/v) fetal bovine serum (Gibco-BRL), penicillin 100 U/ml and streptomycin 100 U/ml and maintained in a humidified atmosphere of 95% air and 5% CO_2_ at 37°C. When the proliferation of the cells was 60–70%, the cells were treated with various concentrations of resveratrol (10, 50, 100 or 150 μM) for 24 h.

### Annexin V/propidium iodide (PI) double staining

Apoptotic cells were quantified using an Annexin V-fluorescein isothiocyanate (FITC)/PI kit (BD Biosciences, San Jose, CA, USA) and detected using flow cytometry using a FACSCalibur™ flow cytometer (Becton, Dickinson and Company, Franklin Lakes, NJ, USA) and analyzed using Modfit and CellQuest™ software (Becton, Dickinson and Company). In brief, cells were pretreated with 10, 50, 100 or 150 μM resveratrol for 24 h and washed with phosphate-buffered saline (PBS). Cells were then collected and resuspended in binding buffer [10 mM 4-(2-hydroxyethyl)-1-piperazineethanesulfonic acid (pH 7.5), 2.5 mM CaCl_2_ and 140 mM NaCl). Cells were incubated with Annexin V-fluorescein isothiocyanate and PI for 15 min in the dark, prior to flow cytometric analysis. Annexin V-positive cells were considered to be in the early stage of apoptosis, whereas Annexin V and PI-positive cells were considered to be in the late stage of apoptosis.

### Cell cycle analysis

Cells were quantified using a Cell Cycle Analysis kit (Beyotime Institute of Biotechnology, Shanghai, China), detected using a FACSCalibur flow cytometer (Becton, Dickinson and Company) and analyzed using Modfit and CellQuest software 6.1 (Becton, Dickinson and Company). In brief, cells were pretreated with 10, 50, 100 or 150 μM resveratrol for 24 h, washed with PBS, then fixed with 70% ethanol for 24 h. Cells were incubated with propyl iodide organism dye for 30 min at 37°C, followed by flow cytometric analysis.

### MTT assay

HCT116 and Caco-2 cell densities were adjusted to 2×10^4^ cells per 100 μl. Cells were seeded onto 96-well plates, which were placed in an incubator overnight to allow for attachment and recovery. In brief, cells were pretreated with 10, 50, 100 or 150 μM resveratrol for 24 h and MTT was then dissolved to a concentration of 5 mg/ml in warm assay medium. A total of 20 μl MTT solution was transferred to each well to yield a final volume of 120 μl/well. Plates were incubated for 4 h at 37°C in 5% CO_2_. Following incubation, supernatants were removed and 150 μl DMSO was added. Plates were then placed on an orbital shaker for 5 min and the absorbance was recorded using the EnSpire™ 2300 Multilabel Plate Reader (PerkinElmer, Inc., Waltham, MA, USA) at 595 nm.

### Western blot analysis of resveratrol-regulated apoptotic proteins and cell cycle proteins

HCT116 and Caco-2 cells were collected following treatment, then lysed in lysis buffer [100 mM Tris-HCl (pH 6.8), 4% (m/v) sodium dodecylsulfonate (SDS), 20% (v/v) glycerol, 200 mM 2-mercaptoethanol, 1 mM phenylmethyl sulfonylfluoride and 1 g/ml aprotinin] for 30 min on ice. The lysates were separated using centrifugation at 4°C for 15 min at 3,913 × g. The total protein concentration in the supernatants was detected by bicinchoninic acid (BCA) assay using a BCA Protein Assay kit (Beyotime Institute of Biotechnology). SDS-PAGE was performed using an 8–15% gradient or standard polyacrylamide gels. Proteins were subsequently transferred to nitrocellulose membranes, which were saturated with 5% milk in TBST (Tris-buffered saline and 1% Tween-20) and incubated with primary antibodies in a diluent overnight at 4°C. Membranes were washed three times with TBST and incubated with goat anti-rabbit IgG-HRP for 1 h, followed by washing four times with TBST. Detection was performed using an Odyssey^®^ Infrared Imaging System (Li-Cor Biosciences, Lincoln, NE, USA).

### Statistical analysis

Data were analyzed using GraphPad Prism 5 (GraphPad Software, Inc., San Diego, CA, USA). Data are presented as the mean ± standard deviation from triplicate experiments performed in a parallel manner unless otherwise stated. Statistical differences were assessed using the Student’s t-test and P<0.05 was considered to indicate a statistically significant difference. Data are representative of at least three independent experiments.

## Results

### Resveratrol inhibits cell proliferation and promotes cell apoptosis

**Figure 2:**
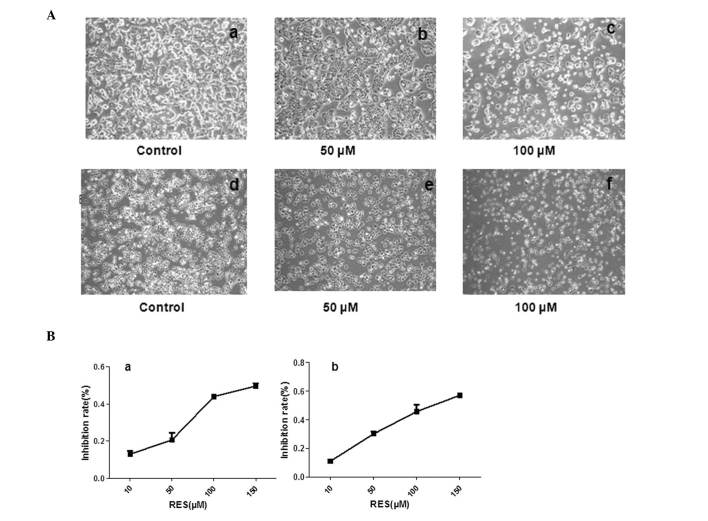
Resveratrol-induced cell proliferation inhibition in (Aa, b and c) HCT116 and (Ad, e and f) Caco-2 cells treated with 50 or 150 μM resveratrol for 24 h, visualized using microscopy (magnification, ×100). (Ba) HCT116 and (Bb) Caco-2 cell growth inhibition rates analyzed using MTT assay. RES, resveratrol.

The untreated HCT116 and Caco-2 cells were observed to be healthy with clear skeletons, whereas the cells treated with resveratrol were distorted with certain cells becoming round. Furthermore, the number of sloughed cells increased with increasing drug concentration (Fig. 2A). MTT assay was used to assess the inhibitory effect of resveratrol on the HCT116 and Caco-2 cells and revealed a significant dose-dependent inhibition of cell growth after 24 h of treatment (Fig. 2B). The IC_50_s for resveratrol on HCT116 and Caco-2 cells were 170 and 120 μM, respectively, which were calculated using GRAFIT-Erithacus IC_50_ software (11). Resveratrol was found to exert a strong inhibitory effect on the viability of HCT116 and Caco-2 cells, which may contribute to its antitumor potency. Cells treated with 50 and 150 μM resveratrol became round and floating, with inhibited cell growth. Furthermore, the majority of the HCT116 and Caco-2 cells underwent severe apoptosis with increasing resveratrol concentration.

**Figure 3:**
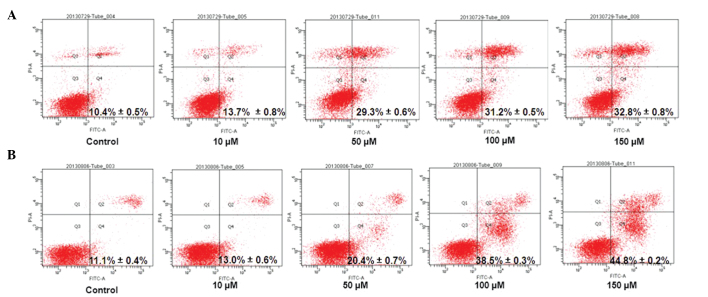
Resveratrol-induced apoptosis in (A) HCT116 and (B) Caco-2 cells treated with various concentrations (10, 50, 100 and 150 μM) of resveratrol for 24 h and analyzed using flow cytometry. Each sample was duplicated and the figure is representative of three independent experiments. Annexin V/propidium iodide stain. Values are presented as the mean ± standard deviation of at least three independent experiments performed in triplicate.

Annexin V/PI double staining was used to detect apoptosis in the HCT116 and Caco-2 cells (Fig. 3). With increasing drug concentration, the apoptosis rates of the cells were found to increase. Resveratrol inhibited proliferation and promoted apoptosis in HCT116 and Caco-2 cells concentration-dependently.

### Resveratrol promotes cell cycle arrest

**Figure 4:**
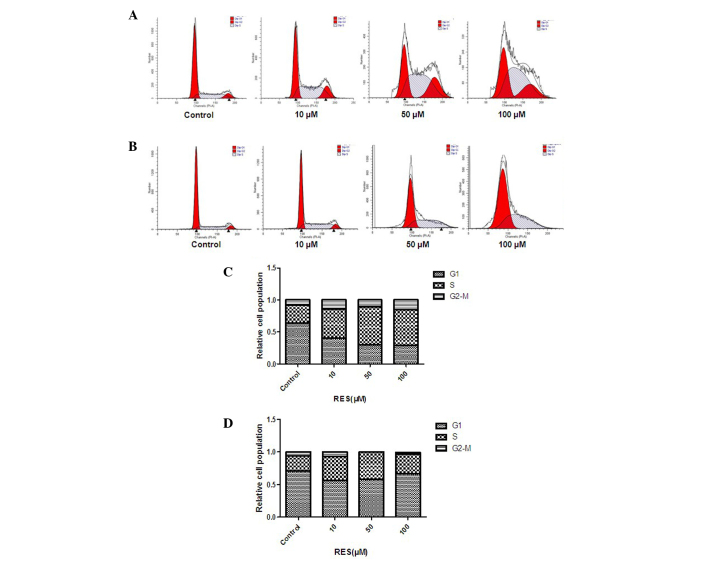
Resveratrol-induced cell cycle arrest in (A) HCT116 and (B) Caco-2 cells treated with various concentrations (10, 50 and 100 μM) of resveratrol for 24 h and analyzed using flow cytometry. (B and D) Cell cycle distributions for A and C. Each sample was duplicated and the figure is representative of three independent experiments. Values are presented as the mean ± standard deviation of at least three independent experiments performed in triplicate. RES, resveratrol.

In order to determine whether resveratrol causes cell cycle arrest in human colon cancer cells, HCT116 and Caco-2 cells treated with DMSO or resveratrol for 24 h were subjected to flow cytometric analysis following DNA staining (Fig. 4). With increasing resveratrol concentration, the proportion of cells in G_1_/S-phase was found to increase after 24 h of resveratrol treatment. These results demonstrated that resveratrol inhibited proliferation and promoted apoptosis in HCT116 and Caco-2 cells in a concentration-dependent manner.

### Resveratrol activates apoptotic proteins and induces cell cycle arrest

**Figure 5:**
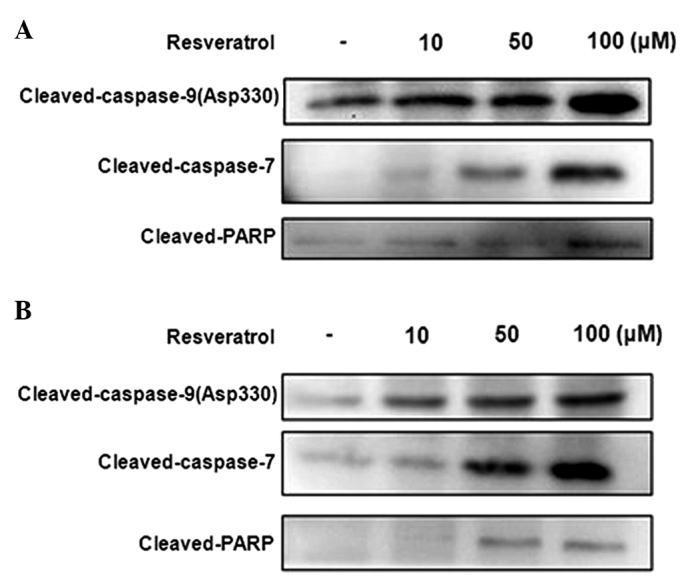
Western blot analysis of the effect of resveratrol on the expression of apoptosis-associated proteins in (A) HCT116 and (B) Caco-2 cells treated with various concentrations (10, 50 and 100 μM ) of resveratrol for 24 h. Western blots are representative of three independent experiments. PARP, poly(ADP-ribose) polymerase.

**Figure 6:**
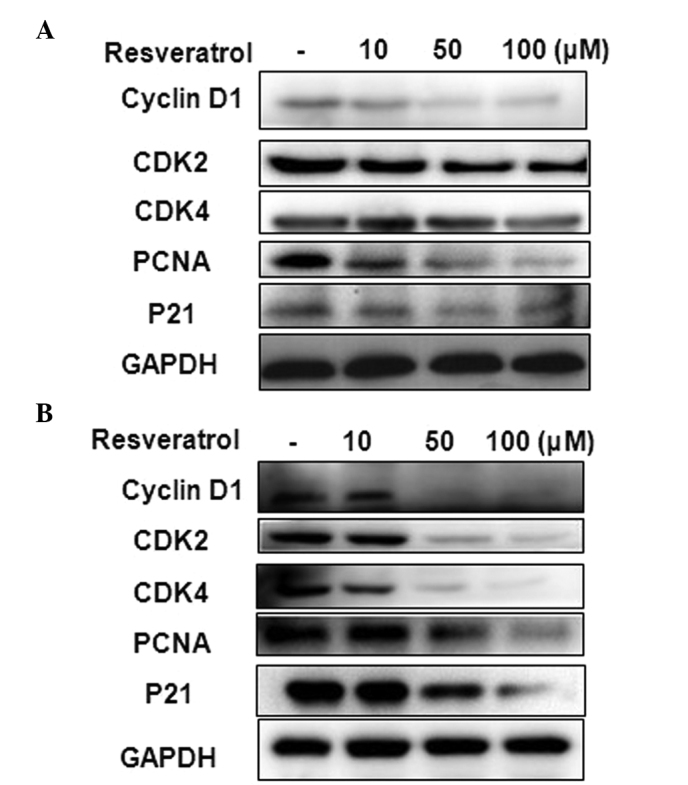
Western blot analysis of the effect of resveratrol on the expression of cell cycle-associated proteins in (A) HCT116 and (B) Caco-2 cells treated with various concentrations (10, 50 and 100 μM ) of resveratrol for 24 h. Western blots are representative of three independent experiments. CDK, cyclin-dependent kinase; PCNA, proliferating cell nuclear antigen.

Cells were treated with 10, 50 or 100 μM resveratrol for 24 h. The apoptotic protein expression of cleaved-caspase-9, cleaved-caspase-7 and cleaved-PARP was found to increase in a concentration-dependent manner in the resveratrol-treated HCT116 (Fig. 5A) and Caco-2 cells (Fig. 5B) compared with the control cells. Furthermore, the protein expression of the cycle arrest proteins CDK2, CDK4, cyclin D1, PCNA and P21 were observed to decrease in a concentration-dependent manner in the resveratrol-treated HCT116 (Fig. 6A) and Caco-2 (Fig. 6B) cells compared with the control cells.

## Discussion

As a stilbene compound, resveratrol has been widely studied due to its health promoting potential (3,12–15). The present study aimed to investigate the effect of resveratrol in HCT116 and Caco-2 human colon cancer cells. In the present study, the inhibitory and apoptosis-promoting effects of resveratrol on the growth of two human colon cancer cell lines with different genetic aberrations and aggressiveness, were assessed. Caco-2 cancer cells were found to be more sensitive to resveratrol treatment than HCT116 cancer cells (Fig. 4). Furthermore, the expression of the apoptosis-associated proteins cleaved caspase-7, cleaved caspase-9 and cleaved PARP (Fig. 5) were found to increase in a dose-dependent manner. The activation of caspase-9 and -7 are crucial steps in apoptotic cell death (16–19), which consequently induce PARP cleavage. PARP has been proposed to be important for controlling numerous cellular processes, including DNA repair, cell death, chromatin function and genome stability (20–22). PARP activation through cleavage is an early DNA damage response. The present study found that resveratrol increased cleaved-caspase-9 and -7 protein expression in HCT-116 and Caco-2 cells, while cleaved PARP, which is a marker of cell apoptosis, was increased.

Furthermore, the present study demonstrated that human colon cancer cells are susceptible to resveratrol-induced inhibition of proliferation, predominantly through cell cycle arrest. Of note, the Caco-2 cells were observed to be more sensitive to resveratrol-induced cell cycle arrest than the HCT116 cells.

Resveratrol has been shown to induce cell cycle arrest in a variety of other human cancer cell lines, including breast cancer, leukemia, prostate and colon cancer (12,24,25). Cell cycle progression, including genome duplication, is controlled by CDKs and CDK inhibitors. Among the various roles reported for p21, the most studied role is associated with its CDK and PCNA binding domains (26,27). CDK genes are often constitutively expressed and CDKs are relatively stable, whereas cyclin genes exhibit periodic patterns of expression and are subject to regulated degradation. During the transition between G_1_- and S-phase, cyclin D1 and CDK4 are the first to be expressed during early G_1_-phase (28). Cell cycle arrest occurs due to the loss of cyclin expression and CDK activity. In order to investigate the mechanism underlying resveratrol-induced G_1_/S-phase cell cycle arrest, cell cycle regulatory proteins and CDKs were analyzed using western blot analysis. In the present study, resveratrol was found to induce G_1_/S-phase cell cycle arrest (Fig. 3). Furthermore, the expression of cyclin D1, CDK4, P21, PCNA and CDK2 were observed to be reduced in a concentration-dependent manner in the HCT-116 and Caco-2 cells (Fig. 6). These findings therefore suggested that the resveratrol-induced G_1_/S-phase cell cycle arrest may be mediated through the cyclin-CDK checkpoint.

In conclusion, the present study identified that resveratrol induced apoptosis and cell arrest in HCT116 and Caco-2 human colon cancer cells through caspase-dependent and cyclin-CDK mechanisms. Furthermore, the Caco-2 cells were observed to be more sensitive to resveratrol treatment than the HCT116 cells. These findings suggested that resveratrol may be a novel candidate for colon cancer therapy.
